# Business Models in Water Supply Companies—Key Implications of Trust

**DOI:** 10.3390/ijerph17082770

**Published:** 2020-04-17

**Authors:** Adam Jabłoński, Marek Jabłoński

**Affiliations:** Faculty in Chorzów, Wyższa Szkoła Bankowa University in Poznań, Sportowa 29, 41-506 Chorzów, Poland; marek.jablonski@ottima-plus.com.pl

**Keywords:** social enterprise, business model, trust, water supply companies

## Abstract

Currently, trust is one of the key factors that ensures the acceptable mechanisms of economic and social relationships. It is not only an element of correct communication, but also a factor in inter-organizational bonds and a source of social dialogue. Trust has become a factor in the creation of value, as well as a key component of the conceptualization and operationalization of business models. It has revealed many problems at the strategic level, in the water sector in particular. From this perspective, trust is a major factor of strategies, models, and business processes which are currently being built. New types of business models that emerge have also started to include trust as part of their configuration. This is the case in the construction and implementation of social business models. A social business model can be understood as a business model whose factors that stimulate development include social aspects expressed in balancing economic, environmental, and social issues with the involvement of communities and their dynamic communication focused on the selected attributes of business models that stimulate growth and that are conducive to achieving success, expressed by economic and/or social profit. The satisfaction of stakeholders with such a solution is another condition for embedding this solution in the sphere of the social economy. In this approach, trust, which stimulates the growth of social and economic value in the component structure of the social business model, becomes particularly important. The aim of the paper is to present the place and role of trust as a key component of social business models. The scope of the paper includes research into public water sector industry companies located in the Province of Silesia and their social business models, with a focus on defining the position of trust among other attributes of these business models. The authors put forward a hypothesis that trust is a crucial component of the social business models of water supply companies that operate at the intersection of the market and social economy. Trust also helps companies from the water supply sector achieve both social and economic effects. It also becomes a source of reverse market polarization, where the value of a social business model materializes to create social and environmental effects without detriment to the economic effects.

## 1. Introduction

The dynamics of the changes taking place in the modern economy are significant. Not only are strategic priorities changing, but the logic of running and understanding business is as well. Many of the principles and management mechanisms thus far considered common have been depleted and sometimes even rejected. This strategic trajectory discloses new factors that determine market development and the various types of organizations located therein. The diverse types of organization and the complexity of the issues of managing them characterize the new shape of the economy. The norms of the classical market economy have become significantly outdated. The impact of various types of communities has resulted in changes, not only in managerial intentions themselves, but above all in the awareness of the business and its key attributes. The change in these attributes has led to a modification in the structure and interfaces in the created business models of modern enterprises. The attributes marked by social elements, which can include trust, have come to the fore. This component plays a special role specifically in social business models, and has become the very component of the business model and a platform for dialogue with various types of communities, including communities operating in a virtual digital environment. Interestingly, it has begun to play a significant role in hybrid organizations, which include water supply companies. In such defined boundary conditions, a scientific problem emerges that is related to the construction of social business models based on the trust of water supply companies. There is also a cognitive gap associated with the lack of sufficient studies on the creation of social business models, their optimal and scalable shape with the definition of the place and role of trust in water supply companies in particular. The aim of the paper is to present a report on in-depth scientific research into the creation of social business models based on trust in water supply companies. The scope and subject of the paper includes scientific research between water supply companies located in a highly urbanized industrial area, in Silesia in Poland. The authors formulate the main hypothesis that trust shapes the social business model of a water supply company. In this understanding of this important issue, the authors direct the scientific discourse to the areas of economics of value associated with the conceptualization and operationalization of social business models.

It should be noted that the presented scientific discussion is related to the interdisciplinary approach to the concept of business models of companies, strategic management in the water supply sector, trust, environmental engineering, industrial ecology and innovative approaches and solutions in the field of technology, and strategic dimensions of the existence of water supply companies and their place or role in the environmental protection sector. 

In the paper, the authors present their own scientific research related to determining the place and role of trust in constructing the business models of water supply companies. The authors are aware that there is a certain scientific gap in this area, which is related to the fact that until now there has been no extensive research in the field of treating trust as a component of the business model. The research primarily concerned trust as a form of strengthening positive relationships and mutual dialogue with key actors in the value chain. So far, there has been no reference to trust as an element of the configuration of the business model with the definition of its key interfaces. This approach seems justified due to the increased use of mechanisms of building and operationalizing business models as an important determinant of the functioning of companies on the market with the ability to create economic and social values through this business model. Hence, it seems that research should be of great importance in the understanding of the principles of the functioning of water supply companies and their key ontological entities, which undoubtedly include the business model and strategy. In addition, water supply companies by definition pursue hybrid goals; therefore, they must simultaneously fulfill social and economic obligations and offer a high-trust product, i.e., water, which is important in the context of considering trust as a component of the business model. Trust in the quality of water and its safe consumption is also a guarantor and a platform for building mutual, positive relationships [1]. Therefore, it seems that some added value will be created, which can be further increased, and which will become a source of scientific inspiration and further research and scientific discussions about this topic.

This paper is structured as follows: Section 1 presents the theoretical context of a social company—a strategic dimension, business models in the social economy and their strategic mechanisms and the problem of trust as an attribute of the social business model. Section 2 presents the adopted methodology of scientific research along with a description of the research sample for the water supply market, an analysis of basic data on the water supply companies surveyed, a description of the research conducted and an explanation of the adopted hypotheses and an analysis of the results. Section 3 presents a discussion on social business models based on trust in water supply companies. In Section 4 and Section 5, conclusions and limitations are presented. Finally, limitations are declared and future research which can be used to develop the topic is indicated. The paper ends with references.

### 1.1. A Social Enterprise—A Strategic Dimension

The market changes currently occurring, which have been caused by many surprising issues, have resulted in a totally different polarization of running a business. Business done only according to the principles of neoclassical economics has ceased to be the only challenge for managers. There has even been a change in the understanding of the company, which is not only a tool for multiplying assets for shareholders, or a resource-based organization, but above all is a social being that satisfies the most sophisticated needs of various groups of stakeholders, who create a specific community located either closer to or further from the company. From this perspective, there has been a shift from a market economy to a value-based social market economy. The main assumption is now not only to run a business, but above all to serve many communities. This, however, has resulted in great difficulty in both the construction and management of such a company. Solutions based on game theory can be used here [2]. It is also important to refer to the social system linked with the economy. The difference between the success and failure of a particular social system could result from individual beliefs about the survival of a marriage—or company—in the future, which determines the direction of putting in the least amount of effort [3].

According to M. Yunus, a social business is different. Operated in accordance with management principles just like a traditional Profit-Maximizing Business (PMB), a social business aims for the recovery of all costs, or more, even as it focuses on creating products or services that provide a social benefit. It pursues this goal by charging a price or fee for the products or services it creates [4]. Social enterprise is a collective term for a range of organizations that trade for a social purpose. They adopt one of a diverse range of legal formats, but have in common the principles of pursuing business-led solutions to achieve social aims, and the reinvestment of surpluses for community benefit [5]. Social entrepreneurs demonstrate the ability to form balanced judgments. G. Sullivan Mort, J. Weerawardena and K. Carnegie argue that they exhibit balanced judgment or a coherent unity of purpose and action in the face of complexity, and that this constitutes the third dimension of the proposed multidimensional construct [6]. The principles on which social economy and social entrepreneurship are founded bring to mind the conception of businesses based on ethical and moral behaviors of the organizational members as well as the community. In turn, such behaviors have consequences in the society in which the businesses are embedded [7]. Social entrepreneurs make significant and diverse contributions to their communities and societies, adopting business models to offer creative solutions to complex and persistent social problems. It is proposed that social entrepreneurship “encompasses the activities and processes undertaken to discover, define, and exploit opportunities in order to enhance social wealth by creating new ventures or managing existing organizations in an innovative manner” [8]. T.H. Roh says that the idea of “social economy or social organization” suggests and enlarges the area to include the creation of company social shared value. Social enterprise operations that are able to create both social and economic value are considered “double bottom-line” organizations, while other public organizations are mainly “single bottom-line” organizations [9]. Social entrepreneurs need to reach adequate levels of compliance with each logic to generate the support needed to survive and thrive. Additionally, given their innovative approach to realizing social goals by economic means, social entrepreneurs not only need to be aware of and enact each logic to satisfy stakeholders’ demands, but also need to be able to internally combine social welfare and commercial logic in order to build sustainable and stable hybrid strategies [10]. In order to fulfill external responsibility prospects, the social impact dimension has become an important practice for social enterprises. However, the ambiguity around social impact and its measurement leads to resistance among stakeholders involved in a social enterprise [11]. It is important to undertake a comprehensive review of the concept of social entrepreneurship, especially in the context of not only understanding it, but above all applying it. The definitions presented below give a definite overview and picture of the understanding of this concept as seen from many cognitive perspectives. Such a review allows for certain strategic reflections on the use of this concept, both to conceptualize and operationalize business and build business models. It is worth noting that these types of organizations often operate in regulated sectors. Efficiency incentives promoted by the regulator have influenced behaviors within the industry, sometimes in counterintuitive ways. For example, water companies are tempted to pursue radical change in supply networks and business models for each program simply to capture innovation-related rewards rather than to obtain resource efficiencies [12]. In addition, this has strong determinants also in sectors related to environmental protection. Environmentally friendly or green behavior is a topic of growing importance for governments, companies, and societies. Engaging consumers in green behavior is a challenge for policymakers and managers who strive for sustainable development [13].

According to Fowler [14], social entrepreneurship is the creation of viable socio-economic structures, institutions, organizations, relations, and practices that yield and sustain social benefits. However, Austin et al. [15] believe that social entrepreneurship is an innovative, social value-creating activity that can occur within or across the nonprofit, business, and public sectors. Social entrepreneurship is a multidimensional construct involving the expression of entrepreneurially virtuous behavior to achieve the social mission, a coherent unity of purpose and action in the face of moral complexity, the ability to recognize social value-creating opportunities and key decision-making characteristics of proactiveness, risk-taking, and innovativeness [6]. Robinson [16] defines social entrepreneurship as a process that includes the identification of a specific social problem and a specific solution, the evaluation of the social impact, the business model, and the sustainability of the venture; and the creation of a social mission-oriented for-profit or a business-oriented nonprofit entity that pursues the double (or triple) bottom line. Social entrepreneurship is a process consisting of the innovative use and combination of resources to explore, which aims to bring about social change by catering to basic human needs in a sustainable manner [17]. Hockerts [18] believes that social purpose business schemes are hybrid enterprises straddling the boundary between the for-profit business world and social mission-driven public and nonprofit organizations. Numerous authors [19,20,21,22] have defined the main building blocks of social entrepreneurship as sociality, innovation, and market orientation:

- socially innovative solutions have been pioneered by social entrepreneurs in terms of employment practices, energy usage, supply chain and recycling, and access to credit and financial services;
- sociality (education, health services, training, economic development, international aid and disaster relief, social justice, political change, environmental planning and management);
- market orientation is most clearly manifested in the for-profit social enterprise form, which operates in commercial markets and creates profits to reinvest in their social mission.

For civil society actors, social entrepreneurship may characterize a driver of systemic social change, a space for new hybrid partnerships, or a model of political transformation and empowerment [23,24,25,26,27,28,29]. Social entrepreneurship includes the activities and processes undertaken to discover, define, and exploit opportunities in order to increase social wealth by creating new ventures or using an innovative method of managing surviving organizations [30]. There are three types of social entrepreneurship [31]:

- “complementary” (where commercial revenues cross-subsidize the social mission of a related not-for-profit);
- “integrated” (when economic activity in itself produces social outcomes);
- “re-interpreted” (when an existing not-for-profit increases its earned income).

When analyzing and identifying the definitions presented, it is worth paying attention to their multidimensionality and complexity as the definitions are created according to different cognitive keys. This definitional ambiguity also delays the implementation of solutions at the enterprise level, so it is also important to review the concept of social entrepreneurs. In this case it also presents a complex and unclear representation, because it also involves the behavior of the entrepreneur himself, who applies value-based business principles from the very managerial intentions to the business decisions implemented. It is also worth noting that the review of these definitions allows for the development of one’s own opinion on the perception of organizational behavior and the decision-making processes of social entrepreneurs. Numerous new opportunities arise. The capabilities approach considers that each person is an end in himself/herself and the quality of his/her life consists of the answer to the question: what can he/she be and do? [32].

Social entrepreneurs are people who realize where there is an opportunity to satisfy some unmet need that the state welfare system will not or cannot meet, and who gather together the necessary resources (money, people, often volunteers, and premises) and use these to “make a difference” [33]. They create innovative solutions to immediate social problems and mobilize the ideas, resources, capacities, and social arrangements required for social changes [34]. Social entrepreneurs are change organizers in society; they pioneer innovation within the social sector through the entrepreneurial quality of a breakthrough idea, their capacity building aptitude, and their ability to precisely demonstrate the quality of the idea and to measure social impacts [35]. Additionally, reputation considered in the context of technological innovation is important here [36]. Most importantly, a social entrepreneur is someone who takes reasonable risk on behalf of the people their organization serves [37]. Risk definition and its skillful management should take into account the entire value chain implemented by the organization, considering social and economic aspects [38].

The definitions of a social enterprise allow us to primarily discriminate its key attributes and the factors describing them. Another critical element of scientific considerations is to understand the very idea of social enterprises. As in the case of the abovementioned definition, it is not about the definitions itself, but about developing a view as to understanding the model itself and its categorization. It is also important to apply them in various business frameworks and short-term and long-term perspectives. By providing a platform for transdisciplinary dialogue, the scenarios disturb existing assumptions, reintroduce social and justice questions to the technical challenges that face the nexus, and build the capacity for sustainable transformation [39].

Social enterprises are private organizations dedicated to solving social problems, serving the disadvantaged, and providing socially important goods which, in their judgment, were not effectively provided by public agencies or private markets [40]. A social enterprise is any private activity conducted in the public interest and organized with an entrepreneurial strategy, but whose main purpose is not the maximization of profit but the achievement of social and certain economic goals, and which has a capacity to bring innovative solutions to the problem of social exclusion and unemployment [41]. Interesting research in this field was conducted in Korea [42]. Dart [43] believes that social enterprise fluctuates from the traditional understanding of the nonprofit organization in terms of norms, structure, strategy, values, and represents a fundamental innovation in the nonprofit sector. They are orthodox businesses with social objectives whose surpluses are principally reinvested for that purpose in the business or in the community, rather than being driven by the need to maximize profit for shareholders and owners [44]. Social enterprise is a collective term for a range of organizations that trade for a social purpose. They adopt one of a variety of different legal formats but have in common the principles of pursuing business-led solutions to achieve social aims, and the reinvestment of surpluses for community benefit [5]. Alter [21] defined three essential models of social enterprise: external (when business activities are an external source of funding for social programs, typically in health or education not-for-profits); embedded (when social programs are inherent in the business activities); and integrated (when social programs edge with business activities). A social enterprise is a specific piece of the market economy, placing its goals and missions outside the market [45]. Based on whether a business has a more market-driven or socially driven mission and whether or not it requires profit, the Social Entrepreneurship Matrix (SEM) combines those factors that most clearly differentiate social entrepreneurism from traditional entrepreneurism [46]. Social enterprises are organizations with an explicit aim of benefitting the community, initiated by a group of citizens and in which the material interest of capital investors is subject to limits. They place a high value on their independence and on economic risk-taking related to ongoing socioeconomic activity [47]. 

When undertaking a broad analysis of the classifications presented, it is essential to look at them in a holistic way that ensures the development of one’s own cognitive reflections, which will create factors describing them that will also allow for the determination of the rules for conducting scientific research into this problem. In addition, a scientific analysis of the content controlled in these definitions should enable us to draw correct scientific conclusions which synthesize this question in both strategic and tactical-operational terms.

### 1.2. Business Models in the Social Economy and Their Strategic Mechanisms

During multidimensional discussion pertaining to the focus of companies on social aspects, it is important to refer to key ontological entities of organizations that determine their existence on the market. Undoubtedly, a business model is currently one such entity. Today, the business model is a factor that in many cases not only determines success on the market, but also becomes a guarantor of survival in the difficult market economy. It is, among others, the configuration of tangible and intangible resources configured together into one coherent system based on the component structure between which there are mutual interfaces. There are many definitions of business models and their interpretation. The approach to the business model as an architecture for product, service, and information flows was presented by P. Timmers [48]. B.W. Wirtz treats a business model as the company’s internal production and incentive system [49]. A business model can also be an abstraction of how business works [50]. A business model depicts the content, structure, and governance of transactions designed so as to create value through the exploitation of business opportunities [51].

A good business model remains indispensable to any organizational success, regardless of whether it is a new venture or a recognized player [52]. A business model is a framework for making money [53] consisting of four interlocking components that, taken together, create and deliver value [54]. A business model is a conceptual tool containing a set of objects, concepts and their relationships with the objective of expressing the business logic of a specific company [55]. Business models are not recipes or role models, but can play any—or all—of these different roles for different firms and for different purposes—and often play multiple roles at the same time [56]. A business model describes the rationale of how an organization creates, delivers, and captures value [57]. A business model articulates the logic and provides data and other evidence that demonstrates how a business creates and delivers value to customers [58]. Often, with regard to the concept of business models, attention is paid to innovation. In this conceptualization, business model innovation occurs when a company adopts a novel approach to commercializing its underlying assets. One arena in which many companies with important knowledge assets are currently innovating is in the rising "markets for technology", where companies sell rights to their intellectual property rather than themselves directly commercializing products and services based on their knowledge capital [59]. Business model innovation describes the design process for establishing a fairly new business model on the market, which is accompanied by an adjustment of the value proposition and/or the value constellation and aims to generate or secure a sustainable competitive advantage [60]. An important approach to understanding the place and role of the concept of business models is to consider them in the context of the social economy, especially in strategic terms. Sustainable business models are a platform for the transition from business models based solely on profit to social business models. The sustainable business model helps describe, analyze, manage, and communicate (i) a company’s sustainable value proposition to its customers and all other stakeholders, (ii) how it creates and delivers this value, (iii) and how it captures economic value while maintaining or regenerating natural, social, and economic capital beyond its organizational boundaries [61]. M. Yunus, B. Moingeon, and L. Lehmann-Ortega believe that social business solves social problems while being financially sustainable [62]. Social businesses in particular address a social need while generating profits typically reinvested into the business itself, but there is limited understanding of the ways through which social businesses achieve scale [63]. Social business models are primarily implemented by social enterprises. Social entrepreneurs make significant and diverse contributions to their communities and societies, adopting business models to offer creative solutions to complex and persistent social problems. S.A. Zahra, E. Gedajlovic, D.O. Neubaum, and J.M. Shulman propose that social entrepreneurship “encompasses the activities and processes undertaken to discover, define, and exploit opportunities in order to enhance social wealth by creating new ventures or managing existing organizations in an innovative manner” [8]. In relation to the above, hybrid forms of the organization appear that simultaneously achieve economic and social goals, which results in hybrid business models being built. Hybrid organizational forms that combine commercial and welfare institutional logic play an increasingly important role in addressing the grand societal challenges faced today. Based on the literature on hybrid organizations and social business models, the characteristics of social businesses are explored from a business model perspective [64]. Trust seems to be the factor connecting the social and economic goals of these enterprises. 

### 1.3. Trust as an Attribute of The Social Business Model

When conducting a multidimensional analysis related to the search for market success factors of enterprises, it is worth noting that they are often not just the so-called hard factors, but also soft strategic mechanisms that determine the growth and development of enterprises. Such soft components include the concept of trust.

According to Y. Huang and I.F. Wilkinson, trust both shapes the behavior taking place and is shaped by it. At any time, the nature and degree of trust in the relationship influences how those complex components act, interact, and react. The experience and outcomes of these actions, interactions, and reactions affect the nature and degree of trust in the relationship through their impact on actors’ goals related to the resources, relationships, and feelings towards and beliefs about each [65]. The consequences support the proposed modeling of the stages and highlight the positive effect of reputation on calculative trust. Conflict communication, resolution, and sympathy positively affect cognitive trust. Interdependence also exists among the three forms of trust, both directly and indirectly. Affective trust also leads directly to greater investments in relationships and generates additional confidential communication. Trust may generate economic gains, but it also creates room for dishonesty and exploitation. Hence, trust does not remove or reduce risk, but rather creates it [66].

That is, calculative trust does not affect investments in relationships or confidential communication, but cognitive trust influences these constructs indirectly, through the mediation of affective trust [67]. Deeper interactions foster the development of eco-innovations as greater frequency and intensity generates trust and copes with both the complexity of the required knowledge and the need for it to reduce the initial cognitive distance between partners [68].

The following is an overview of selected definitions of trust which aim to provide the framework for a scientific problem related to the potential interpretation of this concept perceived in a diverse way by individual scientists. It is mainly aimed at developing a position about understanding the essence of trust and determining strategic assumptions related to the application of trust in building social business models. When studying these definitions, a key attribute developing from the scientific analysis of the content of the definitions presented was identified and indicated in the table.

Behavior is an attribute of trust and it falls between knowledge and ignorance of man (a hypothesis about his behavior) [69]. In [70], according to Hardin, trust consists of three elements: a person who trusts and who is trusted and the relationship between these people (that is, A trusts B to do X). Trust is a relational phenomenon. It always refers to the relationship between specific entities. Trust is an alternative to credibility which, ensured by various social solutions, institutions, or standards, allows one to take actions based on confidence rather than trust. This definition includes the components of relationships and credibility. Trust is an element of social capital that “refers to features of a social organization such as trust, norms and networks that can improve the efficiency of society by facilitating coordinated actions” [71]. Trust depends on the recognition of norms and values commonly shared by the group, as well as the sacrifice or rescheduling of satisfying your needs for the benefit of the group. Sacrifice is an attribute thereof [72]. In [73,74], according to Inglehart, where experience is an attribute, social trust is examined in three dimensions:

- horizontal, private;
- horizontal, generalized;
- vertical-public (in relation to various types of institutions).

The above-defined social trust in three dimensions, namely vertical (towards various types of institutions) and two horizontal—private and generalized—is particularly important in order to understand the concept of trust from a broader perspective. According to Inglehart, vertical trust, which is rational, changes relatively quickly and in a predictable way as a result of new experiences; whereas social trust, which is determined by expectations and feelings of a moral nature, is more difficult to achieve, because cultural changes necessarily progress slower, often over the course of a generation. The following aspects can be distinguished within vertical trust: generalized trust in management and the belief that they act competently and in good faith, trust in the managerial and interpersonal competencies of the supervisor, good relationships with the supervisor, loyalty to the supervisor, and obtaining information from the supervisor regarding current activities. Within horizontal trust, the following factors can be distinguished: kindness, i.e., a belief in the friendliness of colleagues, help or a belief in the possibility of counting on the help of colleagues in various situations, a belief in the competence of colleagues, their reliability, good relationships with colleagues, sharing knowledge in a team and sharing common goals. Within institutional trust, the following factors can be distinguished: the effectiveness of communication in the company, the acceptance of the company’s development directions, coping with conflicts within the company, development conditions created by the company, sense of security in the company and employees’ own commitment, and the commitment of colleagues [75]. The aspects of vertical trust affect the level at which employees feel motivated, among others. Vertical trust refers to the characterized relationship between the superior and subordinate through asymmetry and dependence in terms of promotion, salary increase, or job security [76]. It is worth noting that public trust is vertical, where the trust relationship occurs between partners located hierarchically and unevenly, between the authorities and citizens. On the other hand, horizontal trust occurs in relationships between colleagues. Horizontal trust is built between employees and their immediate supervisor [77] and is the belief that employees’ actions can be relied on [78].

Social trust, which is determined by opportunities and feelings of a moral nature, is more difficult to achieve, because cultural changes are much slower. Distributed trust occurs between people who are separated by relatively large social distance. Interpersonal trust occurs between specific individuals [79], where relationships are an attribute of trust. According to [80], general trust is a second level of social capital. It exists in external networks comprising people from different groups. Generalized trust is one of the dimensions of the so-called social trust, which is the basis of institutional trust. In [81] the first level of trust, the necessary social capital presents strong social networks, connects people who already know each other and have personal (private) trust. They can be combined into closed groups, excluding other individuals. A closed group is a trust attribute. Passive trust is “based on the acceptance of symbols of power established by custom or tradition”. Dynamic trust, which is a mechanism of social solidarity in post-industrial and network societies, is “based on monitoring the honesty of the other person in an open and continuous manner” [82] and has a symbol and honesty as its component. Equivalent of money is another component of trust, which is present in the sharing economy and is a specific complement to money in transactions within the sharing economy, because failure to perform the contract results in a loss of trust, which in turn will limit or even eliminate the possibility of participating [83]. In [84,85], Grabner-Kräuter, Kaluscha and Tanz, trust in the sharing economy is characterized by the following: includes actual participation; deficiency of interpersonal contacts at the initial stage, i.e., indirect relationships play an important role in the virtual community; making decisions on sharing; direct interactions do not accompany transactions. Their attribute is relationships. Sharing as the component of trust is a key feature of the sharing economy that is based more on social trust rather than the anonymous market forces [86]. Risk and relationships in the sharing economy are the driver of the risk of financial losses (i.e., in e-commerce transactions), and the risk of physical harm or risk to one’s life. Trust in relation to users on internet platforms is built on the basis of opinions of other users, each added piece of information, and a complete profile of a given person [87]. Credibility, which is built among internet platform users, includes a combination of several elements that make up the D.R.E.A.M.S. structure:

- declaring personal information (photo, name, surname) in the profile (Declared);
- rating and opinions of other users (Rated);
- financial assurance for the completion of the service (Engaged);
- recording the level of user activity on the platform (Active);
- content verification and limiting public change (Moderated);
- connecting the profile with other accounts on social networks, i.e., Facebook, LinkedIn (Social) [88].

This concept is understood and used differently for different purposes in theory and practice. The definitions of trust presented above clearly indicate that this is a very important concept, and not only in management sciences. It is also worth noting that these definitions not only describe the issue of trust in various ways, but are also built on different methodological foundations.

### 1.4. Trust and the Circular Economy

When conducting a multidimensional analysis of trust in various approaches, it is worth paying attention to the relationship of trust in the context of the increasingly popular circular economy, which has a strong impact on the mechanisms of building social capital in the economy and in individual companies. The circular economy is of great importance in many sectors of the economy and industry, especially because it sets new development directions, particularly in terms of sustainable development. The concept of the circular economy is based on three principles:

Principle 1: Preserve and enhance natural capital by controlling finite stocks and balancing renewable resource flows.

Principle 2: Optimize resource yields by circulating products, components and materials at the highest utility at all times in both technical and biological cycles.

Principle 3: Foster system effectiveness by revealing and designing out negative externalities [89]. 

The European Environment Agency (EEA, 2016) define the key characteristics of CE as follows:

1. less input and use of natural resources;
2. increased share of renewable and recyclable resources and energy;
3. reduced emissions;
4. fewer material losses/residuals;
5. keeping the value of products, components, and materials in the economy [90].

Martin Geissdoerfer et al. even treat the circular economy as a new sustainability paradigm [91]. The circular economy is understood as an umbrella concept (a phenomenon that creates a relationship between pre-existing and independent concepts which aims to develop a regenerative economic system by deliberately slowing, closing, and narrowing the loop of material and energy used [92]. The circular economy (CE) is based on the reconstruction and regeneration of production and consumption systems, which are designed to keep products, components, and materials at their highest usability and value as long as possible within technical and biological cycles [93]. 

Due to feedback occurring therein, the circular economy should be considered in terms of the system. A systematic approach is used not only to achieve goals focused on the efficient use of resources, but also their skillful rotation. This systematic approach should take an assessment of the organization’s life cycle and its business model into account. Life cycle assessment is a “tool for analyzing the environmental burden of products at all stages of their life cycle—from resource extraction, through the production of materials, parts of products and the product itself, as well as the use of the product for post-reject management, through reuse, recycling or final disposal (in effect “from cradle to grave”)” [94]. A systemic approach allows the circular economy to integrate social, economic, and environmental aspects at every level of management:

- micro level—by creating eco-projects, eco-products, minimizing waste, introducing an environmental management system, etc.;
- meso level—by creating industrial eco-parks;
- macro level—the creation of eco-cities, eco-municipalities, and eco-regions [95].

The circular economy aims to separate prosperity from resource consumption, i.e., how to consume goods and services, and thus provide closed loops that prevent the possible storage of used goods in landfills. Production and consumption also involve “transferring pollutants” to the environment at every stage. The literature recognizes the Product Service System for the business model and digital technologies as factors which facilitate the transition towards the circular economy. However, this means serious challenges for companies. In this context, the business models of service systems play an important role as they have been proposed as an opportunity to promote sustainable development [96]. In this sense, the circular economy is a movement towards sustainability. It proposes a system in which reuse and recycling provide substitutes for the use of primary raw materials. Business strategies in the circular economy are limited (non-renewable) and resources are described as:

- product maintenance;
- product reuse and redistribution;
- product revival and regeneration;
- recycling of components and materials from products [97].

This is also related to the entire value chain. Shivam Gupta, Haozhe Chen, Benjamin T. Hazen, Sarabjot Kaur, and Ernesto D.R. Santibañez Gonzalez argue that mutual support and coordination driven by a stakeholder perspective coupled with holistic information processing and sharing along the entire supply chain network can effectively create a basis for achieving the triple bottom line of economic, ecological, and social benefits [98].

By reducing dependence on such resources, the circular economy improves our ability and the ability of future generations to satisfy their needs. The circular economy increases the likelihood of sustainable development [99]. Accenture has defined five types of circular business models:

- input circular models—focused on the possibilities of reusing energy and input material for the process of goods production,
- waste value models—these consist of recovering the resources used in recycling processes, as a result of which waste generated in one production process becomes useful as input material in another production process,
- life expectancy models—aimed at extending the life of products as well as components through actions such as repair, modernization, or resale,
- platform models—based on the efficiency of using products by making them available to a wider group of users (i.e., sharing products).
- product in the service model—focused on offering services instead of selling goods (thus the company remains the owner of the product it is responsible for) [100].

As research into the circular economy in the context of business models of companies shows, the four main modifications indicated above are required to adapt or create a new business model in accordance with the principles of the circular economy. In particular, these modifications require, on the one hand, the implementation of a reverse operation in the supply chain and a higher level of cooperation with supply chain actors, and on the other hand, the creation of new value as part of the proposition for customers, which requires a new way of perceiving purchase processes and a higher degree of cooperation between the company itself and customers [101]. 

An approach based on the effective management of open innovation may be necessary in order to do so. This model integrates the effects of agglomeration, networks, information asymmetries, and trust on open innovation culture [102]. For the development of the open innovation approach to sustainability, it seems to be appropriate and meaningful to emphasize a broader stakeholder approach [103].

It is also important to demonstrate the relationship between innovation capability, innovation type, and company performance [104].

Circular business models have a significant impact on shaping the new dimension of environmental value, among others [105]. New business models based on circularity will require the innovative efforts of multiple stakeholders, such as suppliers, customers, and producers [106]. By extensively reviewing the extant contributions on circular economy through the lens of business model literature, Andrea Urbinati, Davide Chiaroni, and Vittorio Chiesa propose a taxonomy of Circular Economy Business Models based on the degree of adoption of circularity along two major dimensions: (i) the customer value proposition and interface, i.e., the implementation of the circularity concept in proposing value to customers; (ii) the value network, i.e., the ways through which interacting with suppliers and reorganizing changes the own internal activities [101]. It can be assumed that circular business models are a specific variation of sustainable business models, where the resource-based approach plays a key role not only as a source of competitive advantage, but as a source of repeated technological and innovative renewal. Thus, the circular economy is a general strategy for business models focused on the implementation of the assumptions of sustainable development. By closing, narrowing, slowing down, and intensifying dematerializing loops, they lead to the fact that input resources as well as waste and emissions from the organizational system are minimized, which consequently improves the efficiency of sustainable development [107]. 

From this perspective, it is important to determine the relationship between the circular economy and its business models and trust. It seems that if trust is considered one of the components of the business model, this component will become particularly important in relation to circular business models. Trust can be a core component among other components of the circular business model, between which mutual links occur. In addition to treating trust as a component of the circular business model and the dialogue platform, it can also be used as a factor which strengthens the value chain implemented within the circular economy. It can be assumed that these various forms of using trust in the circular economy result in positive relationships primarily between all actors of this circular value network. The outcome of such action may be an increased ecological, economic, and social network effect. At the same time, trust gives the opportunity to permanently multiply these circular cycles towards the total elimination of an adverse impact on the natural environment. Moreover, trust can be a factor which guarantees the safety of participants in the entire value loop as part of the mutual exchange of the individual attributes of this value.

## 2. Research Methodology 

### 2.1. Description of the Adopted Research Sample and Its Location Together with the Justification for the Choice

Research into the place and role of trust in building the business models of water supply companies was conducted in the Province of Silesia in Poland. The Province of Silesia in southern Poland is the largest high-density urbanized industrial area in Central and Eastern Europe. It is also one of the strongest regions in Poland both economically and demographically.

A collective water supply system has existed in Silesia for over 100 years. Throughout the decades, the water supply system has been successfully modernized and expanded, thus covering an ever-larger area and underpinning the specificity of the region. Currently, this water supply system has the highest network density in Poland.

Currently, 24 municipalities belong to the Silesian Metropolis and use between 90% and 100% of the water supply potential of Górnośląskie Przedsiębiorstwo Wodociągów S.A. Katowice. A total of 14 municipalities treat this system only as an additional, back-up source of supply.

Water supply companies located in a highly urbanized industrial area in the Province of Silesia operate mainly on the basis of specific, mutual cooperation based on the conditions of the ring system of the water and sewage sector. The regional consolidation of the water industry and the creation of a common water supply model is currently one of the most important challenges in the region, which means that mutual relationships must be strongly developed both between individual water supply companies and between them and public administration (government and local government). Mutual trust is needed in order to achieve this. 

In technical terms, the length of the main pipelines is over 900 km, supplied from 11 water supply stations. The ring system as a whole allows for continuous water supply to the agglomeration center. Possible interruption of delivery, for any reason, allows for the supply of water from another direction without quantitative limitations. In the event of the failure of any of the main pipelines supplying water from these facilities, water can be fed from the other direction simply by making the necessary adjustment to the main network in this case. Throughout the Province of Silesia, activities are regularly performed to create the best model of water supply to inhabitants in the region. These works guarantee the stability of the water supply to the inhabitants of the Province of Silesia. 

In light of the above, the following justification can be adopted for choosing the research sample and the location of the conducted research:

- The largest urbanized and highly concentrated industrial area in Central and Eastern Europe.
- A water supply system with the highest network density in Poland.
- Specific, mutual cooperation between water supply companies based on the conditions of the ring system of the water and sewage sector.
- In technical terms, the length of the main pipelines is over 900 km, supplied from 11 water supply stations.
- Strongly developed mutual relationships based on trust both between individual water supply companies and between them and public administration (government and local government).

### 2.2. Analysis of Basic Data on the Water Supply Companies Surveyed

In addition to data composed in the field of trust as a key factor in shaping the social business model of the company, the decision was also made to briefly describe the water supply companies surveyed. They were asked for answers pertaining to:

- the number of people employed: 1–9 (micro enterprise), 10–49 (small enterprise), 50–249 (medium-sized enterprise), over 250 (large enterprise),
- forms of ownership: private, public.

**Figure 1:**
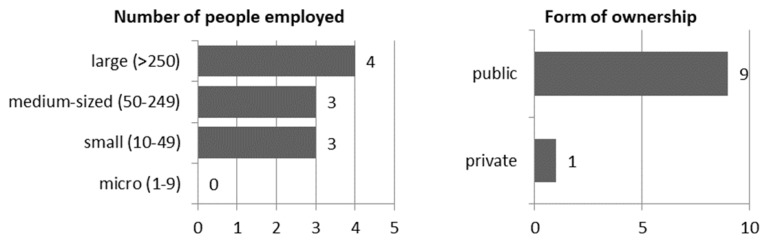
Basic data on water supply companies surveyed.

Among the water supply companies surveyed, 90% public enterprises and 10% were private enterprises. Given the number of employees, distribution was almost even, as 30% of water supply companies employ 10–49 people (a small enterprise); 30% of companies employ between 50 and 249 employees (a medium-sized enterprise); and 40% of companies are large enterprises employing over 250 employees. None of the companies surveyed were a micro-enterprise (employing a maximum of nine employees. Check Figure 1).

One questionnaire template was developed and sent to water supply companies from the Province of Silesia. Answers to 10 survey questionnaires were obtained. All of the survey questionnaires sent were correctly completed according to the requirements. The survey questionnaires were anonymous.

### 2.3. Research Description 

The aim of the paper is to present a report on in-depth research into the structure of trust-based social business models of water supply companies. The scope and subject of the paper includes research on water supply companies located in a highly urbanized industrial area, in Silesia in Poland.

The research phases focus on the following issues: (1) the review and analysis of the appropriate literature covering domestic and foreign references as well as internet sources, (2) the concrete analysis of research and its multidimensional combination aimed at scientific inference, including preliminary research and main research, (3) the development of a research model, (4) the implementation of the analysis and inference process, completed with the development of social business models based on trust in water supply companies in a limited scope, which will be possible to use in the further development of the theory of management science and applicable in the practice of modern business by company managers. The analysis covered the following six issues, based on which questionnaires were developed:

1. Trust-based organizational behavior at the company;
2. Trust-based social capital at the company;
3. Trust-based relationships at the company;
4. Trust-based processes and activities;
5. Trust-based risk at the company;
6. The trust-based business model at the company.

The questionnaires sent to water supply companies contained five statements in each area listed above. The six most important issues were adopted for the analysis, and the appropriate survey statements were constructed (see Table 1):

This model of company analysis can be used outside the water supply industry as well. The issues raised exist in every company and each industry may be subject to similar analysis. It is important because, when developing research statements, a universal approach was applied, which may result in further transferring of the research and its results to other companies located in other sectors.

These are key implications of trust. After the questionnaires were completed, taking into account the above-defined criteria and statements, they were sent to the water supply companies, which were asked to evaluate the criteria in relation to the functioning of their firm and to complete the survey using the following scale (see Table 2).

### 2.4. Description of the Hypotheses

**Figure 2:**
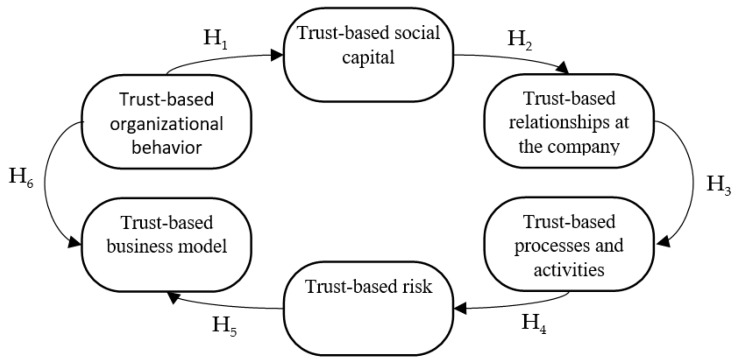
Model of connections within the framework of the research hypotheses.

In order to conduct research in a reliable method and to draw the precise conclusions, the main hypothesis and six supplementary hypotheses were defined. It is also important to determine the relationships between the individual hypotheses (check Figure 2).

Main Hypothesis: Trust shapes the social business model of a water supply company:

- Auxiliary Hypothesis 1. The trust-based organizational behavior of a water supply company affects its trust-based social capital.
- Auxiliary Hypothesis 2. The trust-based social capital of a water supply company shapes its trust-based mutual relations.
- Auxiliary Hypothesis 3. Trust-based relationships in a water supply company determine its trust-based processes and activities.
- Auxiliary Hypothesis 4. Trust-based processes and activities in a water supply company affect its trust-based risk.
- Auxiliary Hypothesis 5. The risk constrained by trust shapes the trust-based social business model of a water supply company.
- Auxiliary Hypothesis 6. Trust-based organizational behavior shapes the trust-based social business model of a water supply company.

The statements least often agreed with included:

- Trust-based risk at the company: Statement 3. At our company, thanks to mutual trust, there are no behaviors that are different from those expected.
- Trust-based business model of the company:
  Statement 1. At our company, trust is a key attribute/component of the business model.
  Statement 2. At our company, trust shapes the business model.

They received the lowest mean rating among the responses. 

The responses received showed that among the responses, the average respondent answered “I somewhat agree” and “I strongly agree”. Out of 30 questions, 12 issues received an average rating of “I strongly agree”. The two highest-rated issues were:

- Trust-based social capital at the company: Statement 1. At our company, trust positively affects the construction of social capital.
- Trust-based relationships in the company: Statement 1. At our company, trust creates mutual relations.

The ratings within each group of criteria were also averaged (see Table 3):

This means that the most important issues in water supply companies are “Social capital at the company”, “Organizational behaviors at the company”, and “Relationships at the company”. The diversity of responses leading to the mean at the level of 4.0 was in the areas of “Risk at the company” and “Company business model”.

### 2.5. Analysis of the Research Results

Based on the data in the table presenting the average results of the rating of companies in terms of social values, the correlation between the responses obtained for each criterion was calculated. Several correlations were presented between the general mean scores obtained from companies within the criteria (C) according to the hypotheses presented at the beginning of the paper. The strength of correlation relationships is defined as:

- 0.9–1.0—the relationship is practically complete;
- 0.8–0.9—very high correlation (very strong relationship);
- 0.4–0.8—moderate correlation (significant relationship);
- 0.2–0.4—low correlation (clear relationship);
- below 0.2—weak correlation (practically no relationship).

**Figure 3:**
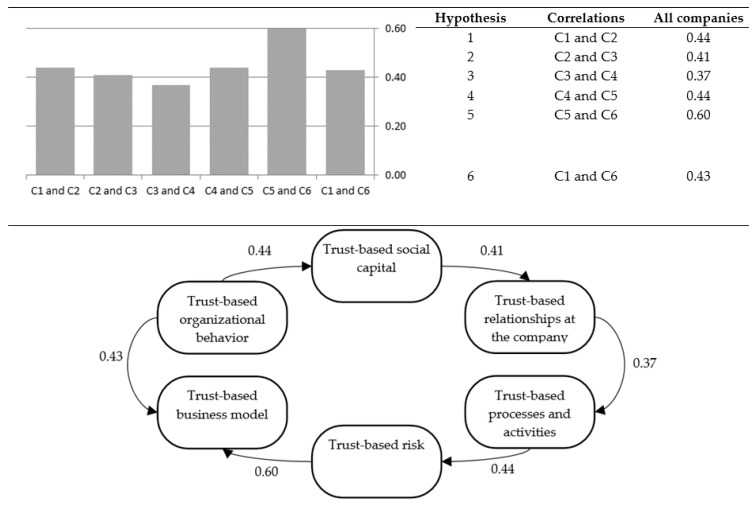
Correlation values between the criteria taking into account in the hypotheses.

The results of these correlations (presented in Figure 3) indicate that the most strongly correlated responses are for Criterion 5 and Criterion 6, with correlation of 0.60 (Hypothesis 5). Thus, in the present study, “5. Risk at the company” and “6. Company business model” have the largest mutual influence. Criterion 3 and Criterion 4 are the least correlated, with correlation at the level of 0.37 (Hypothesis 3). Therefore “3. Relationships at the company” do not have an important impact on “4. Processes and activities in the company” in the model examined. Negative correlation does not occur. 

In order to examine to what extent the data obtained based on the questions and hypotheses affect each other, a decision was made to present this data on the charts and adjust the polynomial trend line function accordingly. In addition, the coefficient of determination *R*^2^ for this trend function was determined. According to the hypotheses, the pairs of variables were analyzed, and the correlations between them were as follows (see Table 4).

In addition, the coefficient of determination *R*^2^ was calculated as the squared values of these correlations. It indicates which part of the total variance of the dependent variable is the part specified by the independent variable. In turn, this shows that this fit is insignificant, as it is mainly at a level below 0.2. In order to determine a better fit within the hypotheses, the trend functions were not linear as before but the exponential functions to the power of 3, and then the fit improved. Standard errors of their structural parameters were calculated for each of the trend functions and recorded in the table below. In order to check whether the model explains the development of the dependent variable to a sufficiently high degree, the coefficient of random variation, coefficient of convergence, and coefficient of determination were calculated (see Table 5).

The coefficient of random variation is calculated using the formula:

$$W_{e}=\frac{S_{e}}{\bar{Y}}\cdot 100\%$$

This formula indicates what percentage of the arithmetic mean of the model dependent variable is the standard abnormality of the recreation. The lower the value of this coefficient, the better the fit. In this study, the lowest value was for Hypothesis 1—the trust-based organizational behavior of a water supply company affects its trust-based social capital, at 4%. The coefficient of random variation *W_e_* reached the highest value (19%) for Hypothesis 6, where the issue of whether trust-based organizational behavior shapes the trust-based social business model of a water supply company was investigated. It means that adjusting the models to the empirical data by the polynomial trend line functions to the power of 3 was appropriate.

The coefficient of convergence was also calculated, which is expressed by the formula:

$$\varphi^{2}=\frac{\sum_{t=1}^{n}e_{t}^{2}}{\sum_{t=1}^{n}{\left(Y_{t}-\bar{Y}\right)}^{2}}$$

This formula indicates which part of the total variance of the dependent variable is not explained by the model. It adopts values from the range [0; 1]. In this analysis, the highest value of the coefficient of convergence ***φ*^2^** was for Hypothesis 3 (trust-based relationships in a water supply company determine its trust-based processes and activities)—as much as 0.86. A not significantly lower value was obtained for Hypothesis 2 (trust-based social capital of a water supply company shapes its trust-based mutual relationships) and amounted to 0.82. However, the smallest value of the coefficient of convergence ***φ*^2^** was for Hypothesis 1 (trust-based organizational behavior of a water supply company affects its trust-based social capital), at the level of 0.18. Due to the fact that the better the fit of the model to the data, the closer the coefficient ***φ*^2^** is to zero, the fit of the models turned out to be high in only one case (Hypothesis 1), moderate in one case (0.43, Hypothesis 4), and low in other cases (Hypotheses 2, 3, 5, 6).

Due to the fact that the following relationship between the coefficient of convergence and the coefficient of determination exists:

$$\varphi^{2}+R^{2}=1.$$

The coefficient of determination *R*^2^ was also calculated. This coefficient says to what extent the total variance of the dependent variable is determined by the independent variables. In this case, the closer the coefficient is to 1, the better the fit. It is expressed by the formula:

$$R^{2}=\frac{\sum_{t=1}^{n}{\left({\hat{Y}}_{t}-\bar{Y}\right)}^{2}}{\sum_{t=1}^{n}{\left(Y_{t}-\bar{Y}\right)}^{2}}$$

Therefore, the best fit was for the function derived for Hypothesis 1, because the value of the coefficient of determination was *R*^2^ = 0.82. The function that was the worst fit was Hypothesis 3, where *R*^2^ = 0.14.

The square root of the coefficient of determination *R*^2^ is a coefficient of multiple correlation. Therefore, after having obtained this data, the decision was also taken to verify the hypothesis about the significance of the coefficient of multiple correlation. This is used to check if the fit of the model to the empirical data is large enough, and the following statistic was used:

$$F=\frac{R^{2}}{1-R^{2}}\;\cdot \;\frac{n-k-1}{k}$$

This statistic has an F Fisher–Snedecor distribution of m_1_ = *k* and m_2_ = *n–k–1* degrees of freedom. The significance level γ = 0.05 and the degrees of freedom m_1_ and m_2_ were determined, and the critical value F* was read from the test tables F (see Table 6).

The value of F statistics was F > F* in only one case, where the coefficient of multiple correlation was significant and the degree of acceptable fit of the model to the data was sufficiently high. In other cases, the coefficient of multiple correlation was insignificantly different from zero (F < F*), and the fit of the model to the data was too weak.

The significance of the structural parameters *αi* of econometric models was also studied to check whether the independent variables significantly affect the dependent variables. The following formula was used:

$$I_{i}=\frac{\left|a_{i}\right|}{S\left(a_{i}\right)},$$

where *a_i_* is the value of the structural parameter evaluation, and *S*(*a_i_*) is the standard error of the structural parameter estimation. The significance level γ = 0.10 and for *n–k–1* degrees of freedom were determined and the critical value I* was read from the Student’s t-test tables. The empirical values of Student’s t-test statistics corresponding to individual structural parameters were calculated (see Table 7).

Only in two cases did the ai structural parameters differ significantly from zero and the independent variable significantly affect the dependent variable. These were:

- Hypothesis 1—the trust-based organizational behavior of a water supply company affects its trust-based social capital;
- Hypothesis 4—trust-based processes and activities in a water supply company affect its trust-based risk.

The probable confidence interval in which the mean is located was estimated, therefore, the confidence interval was determined for the mean of the data obtained. It was assumed that the population variance is unknown, and that *n* < 30, thanks to which the following interval was used to estimate the confidence interval:

$$\left(\bar{X}-t_{\alpha ,\;n-1}\sqrt{\frac{s^{2}}{n}},\;\bar{X}+t_{\alpha ,\;n-1}\sqrt{\frac{s^{2}}{n}}\right)$$

where:

$\bar{X}$—the mean from the sample,
*t_α,n-1_*—distribution function of Student’s t-test distribution for the significance level α and *n-1* degrees of freedom,
*n*—sample size,
$s^{2}=\frac{1}{n-1}\overset{n}{\underset{i=1}{\sum }}{\left(X_{i}-\bar{X}\right)}^{2}$—variance calculated from the sample.

Thus, the following confidence intervals were obtained (see Table 8):

Middle values for individual ranges indicate that the highest value can be expected for the answer to issue 2 (trust-based social capital in the company) from the range (4.47; 4.81). In contrast, the lowest-rated issues were in areas 5 (trust-based risk in the company) and 6 (trust-based business model in the company), for area 5 in the range (3.77; 4.27), and for area 6 (3.71; 4.41). This means that respondents most strongly agreed with the issue of the functioning of social capital in a trust-based company and agreed least with the occurrence of risk-related issues in a trust-based company and the functioning of the business model of a trust-based company. 

The consistency of the scale was verified thanks to Cronbach’s Alpha. This factor takes values from [0; 1] and says to what extent a set of variables is consistent. A reading of α > 0.7 testifies to the high level of reliability of the scale. If all positions were perfectly reliable, the coefficient α = 1. Cronbach’s Alpha was estimated from the following formula:

$$\alpha =\frac{K}{K-1}\;\left(1-\frac{\sum_{i=1}^{K}\sigma_{question_{i}}^{2}}{\sigma_{set}^{2}}\right)$$

where:

*K*—number of questions,
*question_i_*—responses obtained to individual questions as given by all the companies,
$\sigma_{question_{i}}^{2}$*i* variance for the responses that were obtained for a given question *i*,
$\sigma_{\mathrm{set}\;}^{2}$—variance from the sum of responses to all the questions for individual companies.

Cronbach’s Alpha was equal to 0.85. The higher the value of the factor, the greater the consistency of the scale. Consequently, the reliability in this study is very high.

## 3. Discussion

The proposed research model was used to evaluate a complex, multidimensional construct including the principles of building a trust-based social business model of water supply companies in its mechanisms.

The following hypotheses were formulated in the research process.

Main Hypothesis: Trust shapes the social business model of a water supply company:

- Auxiliary Hypothesis 1. The trust-based organizational behavior of a water supply company affects its trust-based social capital.
- Auxiliary Hypothesis 2. The trust-based social capital of a water supply company shapes its trust-based mutual relations.
- Auxiliary Hypothesis 3. Trust-based relationships at a water supply company determine its trust-based processes and activities.
- Auxiliary Hypothesis 4. Trust-based processes and activities at a water supply company affect its trust-based risk.
- Auxiliary Hypothesis 5. The risk constrained by trust shapes the trust-based social business model of a water supply company.
- Auxiliary Hypothesis 6. Trust-based organizational behavior shapes the trust-based social business model of a water supply company.

Not all hypotheses were verified positively. This may indicate that water supply companies in Poland do not fully appreciate the place and role of building the components of trust-based social business models. This may be related to the lack of complete awareness of these issues both in the management processes adopted and in the decision-making processes, among others. It is also worth noting that the companies surveyed do not see the power of influence and logic between management areas such as organizational behavior, social capital, relationships; that is, managerial soft components (see Table 9).

To sum up, it should be noted that social capital is at a relatively low level in Poland due to many political, social, and economic factors. The transformation period had a positive impact on the development of social capital, but there is still a long way to go until standards in this area are equivalent to those of Western European countries. One might even be tempted to say that Poland has a certain deficit of social capital, which is a barrier to the development of companies and their social culture. It is also associated with the certain impoverishment of the Polish society compared to other European countries. Additionally, in recent years, due to changes in the approach to legal and constitutional solutions in Poland, the level of trust of other European countries in Poland has unfortunately decreased significantly. This situation has also resulted in a low level of mutual social trust between companies and between companies and central and local authorities.

On the other hand, the relationship between risk limited by trust, which shapes the trust-based social business model of a water supply company, has a strong influence. However, it is significant that this line of thinking is adopted by water supply companies because many correlations have a moderate correlation (significant dependence). Therefore, it is developmental with an opportunity for a stronger use of these components in the dynamics of the strategic and tactical-operational management of water supply companies.

## 4. Conclusions

Based on the research conducted into the construction of trust-based social business models of water supply companies, a number of conclusions were drawn to identify the assumptions of the theory in this area.

1. The risk limited by trust is much more likely to shape the trust-based social business model of a water supply company.
2. Trust is currently a moderately decisive factor in the social business model of water supply companies in Silesia in Poland.
3. The trust-based organizational behavior of a water supply company moderately affects its trust-based social capital.
4. Trust-based processes and activities at a water supply company moderately affect its trust-based risk.
5. Trust-based relationships at a water supply company moderately determine its trust-based processes and activities.
6. Trust-based organizational behavior moderately shapes the trust-based social business model of a water supply company.
7. The trust-based social capital of a water supply company moderately shapes its trust-based mutual relationships.

When studying the conclusions made, it should be noted that there is potential for further development of water supply companies in these management areas:

- Water supply companies should treat trust on the one hand as an attribute of the business model, and on the other hand as their key success factor, especially in relation to various stakeholders.
- They should intensify activities aimed at increasing the place and role of trust as an essential component of their social business models.
- They should set certain strategic goals in this area and implement them consistently.

## 5. Limitations and Further Directions of Research

By undertaking a multidimensional analysis of the results obtained, the limitations that occurred in the research process were formulated.

1. The research was conducted on a homogeneous test sample of water supply companies, but only in the heavily urbanized area of the Silesia region in Poland.
2. Although survey questionnaires were written in relatively clear language, there is a possibility that not all questions were well understood and interpreted.
3. Survey questionnaires were addressed to top managers; however, the possibility remains that they were filled out by people who did not fully understand the holistic nature of the research conducted.

Summing up the research results, future research directions in this area should be indicated, which can include:

1. Extending research to include the preferences of other stakeholders of the water supply company in this area, and not only the management personnel.
2. Extending research to include other important components of the social business model understood from the perspective of configuration.
3. Examining trust as a key component of the social business model from other cognitive perspectives.
4. Extending research with ranking methods for defining strategic priorities in building the social business models of water supply companies.

The proposals presented do not fully cover the complexity of the study of the processes in question and further needs in this area may be revealed in the course of further research.

## Figures and Tables

**Table 1:** Survey statements.

Trust-based organizational behavior at the company:	Statement 1.1. At our company, trust shapes positive organizational behavior. Statement 1.2. At our company, trust creates positive managerial intentions. Statement 1.3. At our company, employees are characterized by honest and cooperative behavior based on the expressed norms and values. Statement 1.4. At our company, there is an appropriate climate of trust which shapes positive organizational behavior. Statement 1.5. At our company, trust has a special impact on both strategic and tactical-operational behavior.
Trust-based social capital at the company:	Statement 2.1. At our company, trust positively affects the construction of social capital. Statement 2.2. At our company, social capital is based on mutual trust between stakeholders. Statement 2.3. At our company, trust is a key resource for building social capital. Statement 2.4. At our company, trust positively affects the development of employees’ social competences. Statement 2.5. At our company, trust allows us to express the same values, which shape our social capital.
Trust-based relationships at the company:	Statement 3.1. At our company, trust creates mutual relationships. Statement 3.2. At our company, trust improves communication between employees. Statement 3.3. At our company, relationships allow us to apply the same norms of reciprocity. Statement 3.4. At our company, there are continuous interaction and trust-based relationships between employees. Statement 3.5. At our company, trust-based relationships allow us to implement common goals.
Trust-based processes and activities at the company:	Statement 4.1. At our company, trust triggers positive activities. Statement 4.2. At our company, trust allows for less control of processes and activities. Statement 4.3. At our company, trust enables more effective and efficient processes and activities. Statement 4.4. At our company, trust strengthens mutual cooperation both at the level of processes and activities. Statement 4.5. At our company, mutual trust accelerates decision-making processes.
Trust-based risk at the company:	Statement 5.1. At our company, trust is a factor which limits the risk of our activity. Statement 5.2. The reputation of our company is improved thanks to building mutual trust. Statement 5.3. At our company, thanks to mutual trust, there are no behaviors that are different from those expected. Statement 5.4. At our company, trust limits mutual distrust. Statement 5.5. At our company, the risk is mitigated, limited by mutual trust between employees.
The trust-based business model at the company:	Statement 6.1. At our company, trust is a key attribute/component of the business model. Statement 6.2. At our company, trust shapes the business model. Statement 6.3. At our company, trust positively affects other attributes/components of the business model. Statement 6.4. At our company, trust strengthens other components of the business model. Statement 6.5. At our company, thanks to trust embedded in the business model, we achieve a high level of efficiency and effectiveness.

**Table 2:** The scale used to evaluate the company in terms of statements.

Scale	1	2	3	4	5
**Statement**	I strongly disagree	I somewhat disagree	I have no opinion	I somewhat agree	I strongly agree

**Table 3:** Average values of the ratings received for each criterion together with a description.

Criteria	Average Value of the Rating	Description Corresponding to the Rating
2. Social capital at the company	4.6	I strongly agree
1. Organizational behavior at the company	4.4	I somewhat agree
3. Relationships at the company	4.4	I somewhat agree
4. Processes and activities at the company	4.3	I somewhat agree
6. Company business model	4.1	I somewhat agree
5. Risk at the company	4.0	I somewhat agree

**Table 4:** Correlations between hypotheses.

Correlation	C1 and C2	C2 and C3	C3 and C4	C4 and C5	C5 and C6	C1 and C6
	0.44	0.41	0.37	0.44	0.60	0.43
**Coefficient of** **determination *R*^2^**	0.19	0.17	0.14	0.19	0.37	0.19

**Table 5:** Trend functions for the hypotheses and values of the coefficients of random variation, convergence and determination.

Hypothesis	Trend Function	Coefficient of Random Variation *W_e_*	Coefficient of Convergence *φ*^2^	Coefficient of Determination *R*^2^
1	$\begin{array}{c} \hat{X2\;}=249.93-169.62X1+38.74X1^{2}-2.92X1^{3}\\ \left(64.81\right)\left(45.56\right)\left(10.61\right)\left(0.82\right) \end{array}$	4%	0.18	0.82
2	$\begin{array}{c} \hat{X3}=-118.65+82.05X2-18.25X2^{2}+1.35X2^{3}\\ \left(766.67\right)\left(509.39\right)\left(112.34\right)\left(8.23\right) \end{array}$	8%	0.82	0.18
3	$\begin{array}{c} \hat{X4}=22.01-12.99X3+3.07X3^{2}-0.23X3^{3}\;\\ \left(450.7\right)\left(304.12\right)\left(68.19\right)\left(5.08\right) \end{array}$	8%	0.86	0.14
4	$\begin{array}{c} \hat{X5}=-2084.83+1434.08X4-327.58X4^{2}+24.90X4^{3}\\ \left(931.63\right)\left(637.61\right)\left(145.14\right)\left(10.99\right) \end{array}$	11%	0.43	0.57
5	$\begin{array}{c} \hat{X6}=33.48-22.83X5+5.62X5^{2}-0.44X5^{3}\\ \left(114.39\right)\left(85.84\right)\left(21.22\right)\left(1.73\right) \end{array}\;$	17%	0.62	0.38
6	$\begin{array}{c} \hat{X6}=171.20-118.09X1+27.44X1^{2}-2.10X1^{3}\\ \left(260.25\right)\left(182.96\right)\left(42.62\right)\left(3.29\right) \end{array}$	19%	0.76	0.24

**Table 6:** Values of F statistics for the hypotheses.

Hypothesis	Coefficient of Determination *R*^2^	Values of *F* Statistics	Critical Value F*	Conclusion
1	0.82	8.88	4.76	F > F*, the coefficient of multiple correlation is significant and the degree of fit of the model to the data is sufficiently high.
2	0.18	0.44	4.76	F < F*, the coefficient of multiple correlation is insignificantly different from zero, and the fit of the model to the data is too weak.
3	0.14	0.32	4.76	
4	0.57	2.69	4.76	
5	0.38	1.25	4.76	
6	0.24	0.65	4.76	

**Table 7:** Values of I statistics for the hypotheses.

Hypothesis	Value of *I_i_* Statistics	Critical Value I*	Conclusion
1	*I*_1_ = 3.723 *I*_2_ = 3.650 *I*_3_ = 3.568	1.943	The *a_i_* parameter differs significantly from zero and the independent variable X1 has a significant influence on the dependent variable X2.
2	*I*_1_ = 0.161 *I*_2_ = 0.162 *I*_3_ = 0.165	1.943	The *a_i_* structural parameter differs insignificantly from zero, and the independent variable X2 does not significantly influence the dependent variable X3.
3	*I*_1_ = 0.043 *I*_2_ = 0.045 *I*_3_ = 0.046	1.943	The *a_i_* structural parameter differs insignificantly from zero, and the independent variable X3 does not significantly influence the dependent variable X4.
4	*I*_1_ = 2.249 *I*_2_ = 2.257 *I*_3_ = 2.266	1.943	The *a_i_* parameter differs significantly from zero and the independent variable X4 has a significant influence on the dependent variable X5.
5	*I*_1_ = 0.266 *I*_2_ = 0.265 *I*_3_ = 0.254	1.943	The *a_i_* structural parameter differs insignificantly from zero, and the independent variable X5 does not significantly influence the dependent variable X6.
6	*I*_1_ = 0.645 *I*_2_ = 0.644 *I*_3_ = 0.638	1.943	The *a_i_* structural parameter differs insignificantly from zero, and the independent variable X1 does not significantly influence the dependent variable X6.

**Table 8:** Confidence interval for the mean.

Variables	X1	X2	X3	X4	X5	X6
Confidence interval for the mean	(4.20; 4.64)	(4.47; 4.81)	(4.27; 4.57)	(4.17; 4.47)	(3.77; 4.27)	(3.71; 4.41)

**Table 9:** Final conclusions.

Hypothesis	Criterion	Correlation	Conclusion
1	C1 and C2	0.44	This hypothesis showed a moderate correlation between trust-based organizational behavior and trust-based social capital
2	C2 and C3	0.41	This hypothesis showed a moderate correlation between trust-based social capital and relationships in a trust-based company
3	C3 and C4	0.37	This hypothesis showed a low correlation between relationships in a trust-based company and trust-based processes and activities
4	C4 and C5	0.44	This hypothesis showed a moderate correlation between trust-based processes and activities and trust-based risk
5	C5 and C6	0.60	This hypothesis showed a high correlation between trust-based risk and a trust-based business model
6	C1 and C6	0.43	This hypothesis showed a moderate correlation between trust-based organizational behavior and a trust-based business model

## References

[1] Rundblad G., Knapton O., Hunter P.R. (2014). The causes and circumstances of drinking water incidents impact consumer behaviour: Comparison of a routine versus a natural disaster incident. Int. J. Environ. Res. Public Health.

[2] Shi Y. (2019). Economic description of tolerance in a society with asymmetric social cost functions. Econ. Res. -Ekon. Istraživanja.

[3] Zanger I., Padhi S.S., Wagner S.M. (2018). Linking social system failures: A short note on marriage and firm failure. J. Innov. Knowl..

[4] Yunus M. (2008). Creating a world without poverty: Social business and the future of capitalism. Glob. Urban Dev..

[5] Haugh H., Mair J., Robinson J., Hockerts K. (2006). Social enterprise: Beyond economic outcomes and individual returns. Social Entrepreneurship.

[6] Mort G.S., Weerawardena J., Carnegie K. (2003). Social entrepreneurship: Towards conceptualization. Int. J. Nonprofit Volunt. Sect. Mark..

[7] Toledano N. (2011). Social entrepreneurship: The new narrative for the practice of the social economy. CIRIEC-España. Rev. De Econ. Pública Soc. Y Coop..

[8] Zahra S.A., Gedajlovic E., Neubaum D.O., Shulman J.M. (2009). A typology of social entrepreneurs: Motives, search processes and ethical challenges. J. Bus. Ventur..

[9] Roh T.H. (2016). The sharing economy: Business cases of social enterprises using collaborative networks. Procedia Comput. Sci..

[10] Pache A., Chowdhury I. (2012). Social Entrepreneurs as Institutionally Embedded Entrepreneurs: Toward a New Model of Social Entrepreneurship Education. Acad. Manag. Learn. Educ..

[11] Molecke G., Pinkse J. (2017). Accountability for social impact: A bricolage perspective on impact measurement in social enterprises. J. Bus. Ventur..

[12] Li Q., Wang S., Shaw N., Shi V. (2019). Supply Chain Partner Communication in a Managed Programme in the UK Water Industry: A Case Study with Social Network Analysis. Int. J. Environ. Res. Public Health.

[13] Rey-Moreno M., Medina-Molina C. (2020). Dual models and technological platforms for efficient management of water consumption. Technol. Forecast. Soc. Chang..

[14] Fowler A. (2000). NGDOs as a moment in history: Beyond aid to social entrepreneurship or civic innovation?. Third World Q..

[15] Austin J.E., Stevenson H., Wei-Skillern J. (2003). Social Entrepreneurship and Commercial Entrepreneurship: Same, Different or Both?.

[16] Robinson J.A. (2004). An Economic Sociology of Entry Barriers: Social and Institutional Entry Barriers to Inner City Markets. Unpublished Ph.D. Thesis.

[17] Mair J., Marti I. (2004). Social Entrepreneurship: What Are We Talking About? A Framework for Future Research.

[18] Hockerts K. (2004). Bootstrapping social change: Towards an evolutionary theory of social entrepreneurship.

[19] Nicholls A., Cho A.H., Nicholls A. (2006). Social Entrepreneurship: The Structuration of a Field. Social Entrepreneurship.

[20] Stone M., Cutcher-Gershenfeld S., Flynn P., Hodgkinson V.A. (2001). Challenges of Measuring Performance in Nonprofit Organizations. Measuring the Impact of the Nonprofit Sector.

[21] Alter S.K., Nicholls A. (2006). Social Enterprise Models and Their Mission and Money Relationships. Social Entrepreneurship.

[22] Defourny J., Borzaga C., Defourny J. (2001). From Third Sector to Social Enterprise. The Emergence of Social Enterprise.

[23] Nicholls A., Nicholls A. (2006). Introduction. Social Entrepreneurship.

[24] Austin J.E., Leonard H.B., Reficco E., Wei-Skillern J., Nicholls A. (2006). Social Entrepreneurship: It Is For Corporations, Too. Social Entrepreneurship.

[25] Alvord S.H., Brown L.D., Letts C.W. (2004). Social Entrepreneurship and Societal Transformation: An Exploratory Study. J. Appl. Behav. Sci..

[26] Leadbeater C. (1996). The Rise of the Social Entrepreneur.

[27] Nyssens M. (2006). Social Enterprise. At the Crossroads of Market, Public Policies and Civil Society.

[28] Karamchandani A., Kubzansky M., Frandano P. (2009). Emerging Markets, Emerging Models.

[29] Freireich J., Fulton K. (2009). Investing For Social and Environmental Impact: A Design for Catalyzing an Emerging Industry.

[30] Zahra S.A., Rawhouser H.N., Bhawe N., Neubaum D.O., Hayton J.C. (2008). Globalization of Social Entrepreneurship Opportunities. Strategic Management Society.

[31] Huybrechts B., Nicholls A. (2012). Social Entrepreneurship: Definitions, Drivers and Challenges. Social Entrepreneurship and Social Business.

[32] Sanz Ponce R., Peris Cancio J.A., Escámez Sánchez J. (2018). The capabilities approach and values of sustainability: Towards an inclusive Pedagogy. J. Innov. Knowl..

[33] Thompson J., Alvy G., Lees A. (2000). Social entrepreneurship—A new look at the people and the potential. Management Decision..

[34] Alvord S.H., Brown L.D., Letts C.W. (2003). Social Entrepreneurship: Leadership that Facilitates Societal Transformation an exploratory study.

[35] Mair J., Robinson J., Hockerts K. (2006). Social Entrepreneurship.

[36] Höflinger P.J., Nagel C., Sandner P. (2018). Reputation for technological innovation: Does it actually cohere with innovative activity?. J. Innov. Knowl..

[37] Brinckerhoff P.C. (2009). Mission-Based Management: Leading Your Not-for-Profit in the 21st Century.

[38] Đurić G., Todorović G., Đorđević A., Borota Tišma A. (2019). A New Fuzzy Risk Management Model for Production Supply Chain Economic and Social Sustainability. Econ. Res. -Ekon. Istraživanja.

[39] Hoolohan C., McLachlan C., Larkin A. (2019). ‘Aha’ moments in the water-energy-food nexus: A new morphological scenario method to accelerate sustainable transformation. Technol. Forecast. Soc. Chang..

[40] Dees J.G. (1994). Social Enterprise: Private Initiatives for the Common Good. Harvard Business Review.

[41] OECD (2003). The Non-profit Sector in a Changing Economy.

[42] Kim J. (2018). Are countries ready for the new meso revolution? Testing the waters for new industrial change in Korea. Technol. Forecast. Soc. Chang..

[43] Dart R. (2004). The legitimacy of social enterprise. Nonprofit Manag. Lead..

[44] Harding R. (2004). Social enterprise: The new economic engine?. Bus. Strategy Rev..

[45] Hausner J., Laurisz N., Mazur S., Hausner J. (2007). Przedsiębiorstwo społeczne—Konceptualizacja. Zarządzanie Podmiotami Ekonomii Społecznej.

[46] Masseti B.L. (2008). The social entrepreneurship matrix as a “tipping point” for economic change. Emerg. Complex. Organ..

[47] Defourny J., Nyssens M. (2012). The Emes Approach of Social Enterprise in a Comparative Perspective.

[48] Timmers P. (1998). Business models for electronic markets. Electron. Mark..

[49] Wirtz B.W. (2000). Electronic Business.

[50] Eriksson H.-E., Penker M. (2000). Business Modeling with UML: Business Patterns at Work.

[51] Amit R., Zott C. (2001). Value creation in e-business. Strateg. Manag. J..

[52] Magretta J. (2002). Why business models matter. Harv. Bus. Rev..

[53] Afuah A., Tucci C.L. (2003). Internet Business Models and Strategies.

[54] Johnson M.W., Christensen C.M., Kagermann H. (2008). Reinventing your business model. Harv. Bus. Rev..

[55] Osterwalder A., Pigneur Y., Tucci C.L. (2005). Clarifying business models: Origins, present, and future of the concept. Commun. Assoc. Inf. Syst..

[56] Baden-Fuller C., Morgan M.S. (2010). Business models as models. Long Range Plan..

[57] Osterwalder A., Pigneur Y. (2010). Business Model Generation: A Handbook for Visionaries, Game Changers, and Challengers.

[58] Teece D.J. (2010). Business models, business strategy and innovation. Long Range Plan..

[59] Gambardella A., McGahan A.M. (2010). Business-model innovation: General purpose technologies and their implications for industry structure. Long Range Plan..

[60] Wirtz B.W. (2011). Business Model Management: Design—Instrumente—Erfolgsfaktoren.

[61] Schaltegger S., Lüdeke-Freund F., Hansen E.G. (2016). Business models for sustainability: A co-evolutionary analysis of sustainable entrepreneurship, innovation, and transformation. Organ. Environ..

[62] Yunus M., Moingeon B., Lehmann-Ortega L. (2010). Building social business models: Lessons from the Grameen experience. Long Range Plan..

[63] Bocken N.M.P., Fil A., Prabhu J. (2016). Scaling up social businesses in developing markets. J. Clean. Prod..

[64] Spieth P., Schneider S., Clauß T., Eichenberg D. (2019). Value drivers of social businesses: A business model perspective. Long Range Plan..

[65] Huang Y., Wilkinson I.F. (2013). The dynamics and evolution of trust in business relationships. Ind. Mark. Manag..

[66] Reiersen J. (2019). Exchange networks, markets and trust. Econ. Res. -Ekon. Istraživanja.

[67] Akrouta H., Fall Diallo M. (2017). Fundamental transformations of trust and its drivers: A multi-stage approach of business-to-business relationships. Ind. Mark. Manag..

[68] González-Moreno Á., Triguero Á., Sáez-Martínez F.J. (2019). Many or trusted partners for eco-innovation? The influence of breadth and depth of firms’ knowledge network in the food sector. Technol. Forecast. Soc. Chang..

[69] Simmel G. (1975). Socjologia.

[70] Hardin R., Braithwaite V., Levi M. (1988). Trust in government. Trust and Governance.

[71] Putnam R.D. (1995). Demokracja w Działaniu. Tradycje Obywatelskie we Współczesnych Włoszech.

[72] Fukuyama F. (1997). Zaufanie. Kapitał Społeczny a Droga do Dobrobytu.

[73] Inglehart R. (1997). Modernization and Postmodernization. Cultural, Economic and Political Change in 43 Societies.

[74] Inglehart R., Warren M.E. (1999). Trust, well-being and democracy”. Democracy and Trust.

[75] Lewicka D., Krot K. (2014). Zaufanie organizacyjne jako czynnik kreujący proinnowacyjny klimat w Organizacji. Acta Univ. Lodz. Folia Oeconomica.

[76] Sitkin S.B., Roth N.L. (1993). Explaining the Limited Effectiveness of Legalistic “Remedies” for Trust/Distrust. Organ. Sci..

[77] Dirks K.T., Ferrin D.L. (2002). Trust in Leadership: Meta-Analytic Findings and Implications for Research and Practice. J. Appl. Psychol..

[78] Hoe L.S. (2007). Shared Vision: A Development Tool for Organizational Learning. Dev. Learn. Organ. Int. J..

[79] Williams B., Gambetta D. (2000). Formal structures and social reality. Trust: Making and breaking cooperative relations. Trust: Making and Breaking Cooperative Relations, Electronic Edition.

[80] Putnam R.D. (2000). Bowling Alone: The Collapse and Revival of American Community.

[81] Szreter S., Woolcoc M. (2004). Heath by Association? Social Capital, Social Theory, and the Political Economy of Public Health.

[82] Giddens A. (2009). Europa w Epoce Globalnej.

[83] Botsman R., Rogers R. (2010). What’s Mine Is Yours. The Rise of Collaborative Consumption.

[84] Tanz J. (2014). How Airbnb and Lyft Finally Got Americans to Trust Each Other. www.wired.com.

[85] Grabner-Kräuter S., Kaluscha E.A. (2008). Consumer trust in electronic commerce: Conceptualization and classification of trust building measures. Trust and New Technologies: Marketing and Management on the Internet and Mobile Media.

[86] Rifkin J. (2016). Społeczeństwo Zerowych Kosztów Krańcowych. Internet Przedmiotów. Ekonomia Współdzielenia. Zmierzch Kapitalizmu.

[87] Kamal P., Chen J.Q. Trust in sharing economy. Proceedings of the 20th Pacific Asia Conference on Information Systems.

[88] Mazzella F., Sundararajan A. (2016). Entering the Trust Age.

[89] Ellen MacArthur Foundation (2015). Growth within: A Circular Economy Vision for a Competitive Europe.

[90] European Environment Agency (2016). Circular Economy in Europe. Developing the Knowledge Base.

[91] Geissdoerfer M., Savaget P., Bocken N.M.P., Hultink E.J. (2017). The Circular Economy e a new sustainability paradigm?. J. Clean. Prod..

[92] de Pádua Pieroni M., Pigosso D.C.A., McAloone T.C. (2018). Sustainable qualifying criteria for designing circular business models. Procedia CIRP.

[93] Heyes G., Sharmina M., Mendoza J.M.F., Gallego-Schmid A., Azapagic A. (2018). Developing and implementing circular economy business models in service-oriented technology companies. J. Clean. Prod..

[94] Guinée J.B. (2002). Handbook on Life Cycle Assessment: Operational Guide to the ISO Standards. Book Review: The Second Dutch LCA-Guide.

[95] Geng Y., Doberstein B. (2008). Developing the Circular Economy in China: Challenges and Opportunities for Achieving ‘Leapfrog Development’. Int. J. Sustain. Dev. World Ecol..

[96] Bressanelli G., Adrodegari F., Perona M., Saccani N. (2018). The role of digital technologies to overcome Circular Economy challenges in PSS Business Models: An exploratory case study. Procedia CIRP.

[97] Søgaard Jørgensen M., Remmen A. (2018). A methodological approach to development of circular economy options in businesses. Procedia CIRP.

[98] Gupta S., Chen H., Hazen B.T., Kaur S., Santibañez Gonzalez E.D.R. (2019). Circular economy and big data analytics: A stakeholder perspective. Technol. Forecast. Soc. Chang..

[99] Sauvé S., Bernard S., Sloan P. (2016). Environmental sciences, sustainable development and circular economy: Alternative concepts for trans-disciplinary research. Environ. Dev..

[100] Lacy P., Keeble J., McNamara R., Rutqvist J., Haglund T., Cui M., Buddemeier P. (2014). Circular Advantage: Innovative Business Models and Technologies to Create Value in a World without Limits to Growth.

[101] Urbinati A., Chiaroni D., Chiesa V. (2017). Towards a new taxonomy of circular economy business models. J. Clean. Prod..

[102] Nestle V., Täube F.A., Heidenreich S., Bogers M. (2019). Establishing open innovation culture in cluster initiatives: The role of trust and information asymmetry. Technol. Forecast. Soc. Chang..

[103] Rauter R., Globocnik D., Perl-Vorbach E., Baumgartner R.J. (2019). Open innovation and its effects on economic and sustainability innovation performance. J. Innov. Knowl..

[104] Rajapathirana R.P.J., Hui Y. (2018). Relationship between innovation capability, innovation type, and firm Performance. J. Innov. Knowl..

[105] Manninen K., Koskela S., Antikainen R., Bocken N., Dahlbo H., Aminoff A. (2018). Do circular economy business models capture intended environmental value propositions?. J. Clean. Prod..

[106] Chiappetta Jabbour C.J., Lopes de Sousa Jabbour A.B., Sarkis J., Godinho Filho M. (2019). Unlocking the circular economy through new business models based on large-scale data: An integrative framework and research agenda. Technol. Forecast. Soc. Chang..

[107] Geissdoerfer M., Morioka S.N., Monteiro de Carvalho M., Evans S. (2018). Business models and supply chains for the circular economy. J. Clean. Prod..

